# TPP-Based Microfluidic Chip Design and Fabrication Method for Optimized Nerve Cells Directed Growth

**DOI:** 10.34133/cbsystems.0095

**Published:** 2024-05-09

**Authors:** Menghua Liu, Anping Wu, Jiaxin Liu, Yanfeng Zhao, Xinyi Dong, Tao Sun, Qing Shi, Huaping Wang

**Affiliations:** ^1^ Intelligent Robotics Institute, School of Mechatronical Engineering, Beijing Institute of Technology, Beijing 100081, China.; ^2^ School of Medical Technology, Beijing Institute of Technology, Beijing 100081, China.; ^3^ Beijing Advanced Innovation Center for Intelligent Robots and Systems, Beijing Institute of Technology, Beijing 100081, China.; ^4^ Key Laboratory of Biomimetic Robots and Systems (Beijing Institute of Technology), Ministry of Education, Beijing 100081, China

## Abstract

Microfluidic chips offer high customizability and excellent biocompatibility, holding important promise for the precise control of biological growth at the microscale. However, the microfluidic chips employed in the studies of regulating cell growth are typically fabricated through 2D photolithography. This approach partially restricts the diversity of cell growth platform designs and manufacturing efficiency. This paper presents a method for designing and manufacturing neural cell culture microfluidic chips (NCMC) using two-photon polymerization (TPP), where the discrete and directional cell growth is optimized through studying the associated geometric parameters of on-chip microchannels. This study involves simulations and discussions regarding the effects of different hatching distances on the mold surface topography and printing time in the Describe print preview module, which determines the appropriate printing accuracy corresponding to the desired mold structure. With the assistance of the 3D maskless lithography system, micron-level rapid printing of target molds with different dimensions were achieved. For NCMC with different geometric parameters, COMSOL software was used to simulate the local flow velocity and shear stress characteristics within the microchannels. SH-SY5Y cells were selected for directional differentiation experiments on NCMC with different geometric parameters. The results demonstrate that the TPP-based manufacturing method efficiently constructs neural microfluidic chips with high precision, optimizing the discrete and directional cell growth. We anticipate that our method for designing and manufacturing NCMC will hold great promise in construction and application of microscale 3D drug models.

## Introduction

Cell growth regulation is one of the most promising methods in biomedical research such as tissue engineering [1], cancer research [2], and neuroscience [3]. As a typical cell growth regulation method, the directional growth of nerve cells has special significance for neurological medical research, especially for mechanism [4], intervention strategies [5], and drug testing [6] research of central nervous system (CNS) diseases (such as Alzheimer's disease, Huntington’s disease, and Parkinson’s disease). The structure and function of the CNS are affected by the precision and accuracy of nerve cell connections, which may lead to dysfunction [7,8]. Therefore, it is necessary to control the directional growth of nerve cells to help repair or replace damaged cells and promote the repair and regeneration of the nervous system. Common strategies for regulating neural cell growth in vitro include microcontact printing [9], microfluidic culture [10], scaffold [11], spheroids [12], 3D bioprinting [13], organoids [14], etc. However, most of the in vitro neural cell growth regulation methods still have limitations in various aspects, including: (a) the inability to physically isolate different functional units (such as neuronal networks and vascular networks), (b) the unique differentiation characteristics of nerve cells are not considered, and (c) no dynamic mechanical stress is applied.

Among all methods, microfluidic culture is most likely to overcome the above limitations and provide a targeted experimental platform for regulating nerve cell growth. Microfluidic chip is highly customizable and can effectively physically isolate different functional units with high efficiency to simulate organ-level in vivo structures [15,16]. Microfluidic chip can greatly reduce the number of cells and cell culture medium volume used in cell culture, effectively reducing culture costs and contamination risks, and has a high degree of spatial and temporal resolution, which is exactly necessary for the in vitro culture of neural cells [17,18]. Microfluidic chip can accurately simulate dynamic and complex mechanical and biochemical environments at the microscale, affecting cell development and function [19,20]. In addition, microfluidic chips also have good biocompatibility, optical transparency, and electrical insulation, providing strong support for biological experiments and system integration [21]. However, most of the current microfluidic platforms used for neural cell biology research are processed with 2D photolithography [22,23]. This technology is not only less efficient but also has a fixed structure, which greatly limits the diversity of research platforms and the efficiency of manufacturing. Therefore, it is still necessary to efficiently construct neural microfluidic chips with high-precision complex 3D structures based on new engineering technologies.

Additive manufacturing (3D printing) technology creates precision 3D structures at the micrometer or submicrometer scale by depositing materials layer by layer, providing new possibilities to design, customize and rapidly manufacture neural microfluidic chips with multilevel, multiscale, and complex 3D structures [24]. Two-photon polymerization (TPP) lithography, as a typical 3D printing technology, allows real-time control of light intensity and polymerization speed and can achieve precise control of complex structures [25]. For example, Harberts et al. [26] constructed a functional human iPSC-derived neuron network through TPP printing of customized interconnected high-altitude cavity microchannels. Huang et al. [27] proposed 3D hydrogel scaffolds with L929 cells encapsulation fabricated by TPP, and L929 cells occupied the 3D scaffolds after 4 d of incubation. Although TPP can provide a complex and precision cell growth platform, the mismatch between experimental platform structure and cell growth characteristics may lead to cell subdifferentiation and adaptation, limiting the cell's growth performance and developmental potential, such as incomplete maturation or functional achievement of cellular axons. Moreover, due to the particularity of the fluid environment of microfluidic chips, it is necessary to consider the impact of changes in fluid shear stress caused by rapid local dynamic evolution characteristics on cell activity and differentiation processes. Some studies have shown that cell growth is sensitive to the dimensions and internal structure of the microchannel [28,29]. However, the high cost and limited sustained cell activity of neurons limit the scale and feasibility of long-term stable in vitro neural model construction research. Compared with PC12 cells, the SH-SY5Y cell line has the advantage that it is derived from human bone marrow and is similar to human cells in morphology and function. It can be differentiated into neuron-like cells, displaying a range of highly relevant neurobiological characteristics. Moreover, it offers advantages such as ease of large-scale expansion, repeatability, cost-effectiveness, and the absence of ethical concerns compared to primary neuron culture. As a result, it finds extensive application in experimental research in vitro on CNS diseases [30]. Therefore, considering the high efficiency and precision of TPP printing, the design and manufacturing methods of microfluidic chips based on TPP are of great value in neurobiological research such as regulating nerve cell growth.

In this article, we propose a method for designing and manufacturing neural cell culture microfluidic chips (NCMC) based on TPP to optimize the discrete and directional growth of cells and explore the geometric parameters of its corresponding on-chip microchannels. Based on the Describe print preview module and 3D maskless lithography system, micron-level rapid printing of target mold is achieved. The local flow velocity and shear stress characteristics on NCMC with different geometric parameters were simulated. The on-chip microchannel internal structure with minimal local flow velocity and shear stress was identified to effectively protect the cell differentiation process and reduce damage to axons. SH-SY5Y cells with neural differentiation characteristics were selected, and their directional differentiation experiments on NCMC with varying geometric parameters were conducted. The results demonstrate that the TPP-based manufacturing method can efficiently and precisely fabricate NCMCs while optimizing the discrete and directional growth of axons. Furthermore, the future prospects and challenges of applying this approach to more complex biomimetic medical research were also discussed.

## Materials and Methods

### Experimental design

To optimize the discrete and directional growth of nerve cells within microfluidic chips, the growth characteristics during the differentiation of nerve cells under the influence of differentiation-inducing factors were first analyzed to lay the experimental foundation. Prior to conducting cell experiments, molds for NCMC with 3D morphology were fabricated using IP-S photoresist through a 3D maskless lithography system. Utilizing the soft photolithography method, flexible polymers like polydimethylsiloxane (PDMS) are applied to coat molds to form a microfluidic chip with a specific internal structure to meet the needs of nerve cell biology research. Considering the differentiation and growth characteristics of nerve cells, the NCMCs with different microchannel dimensions were designed and manufactured. Within NCMC, interstitial flow is induced through the deliberate control of hydrostatic pressure, facilitating the discrete and directional growth of cells within the 3D microchannels. Furthermore, specific hydrostatic pressure gradients are established at both ends of microchannels of varying dimensions to simulate microfluidic effects at different velocities. Building upon this foundation, on-chip cell culture experiments were conducted using SH-SY5Y cells as a representative example. Cell activity, cell penetration rate, and cell leakage rate were comparatively analyzed to determine the most suitable geometric parameters of the chip structure for achieving discrete and directional cell growth.

**Fig. 1.**
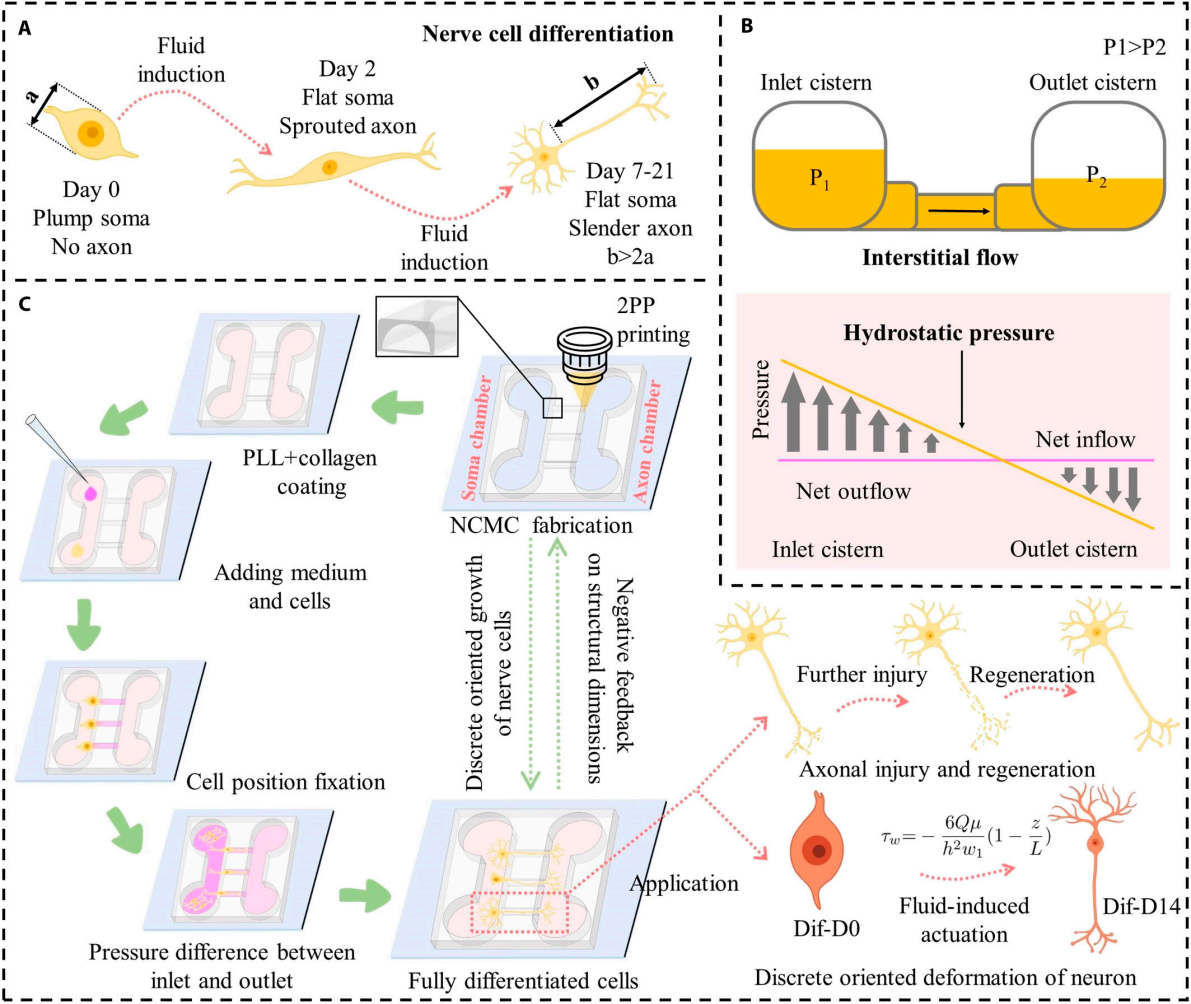
Schematic of the two-photon polymerization (TPP)-based method for optimizing the nerve cell growth in the microchannels of nerve cell culture microfluidic device (NCMC). (A) Differentiation process of nerve cells. (B) Interstitial flow caused by hydrostatic pressure drives the deformation of differentiable cells. (C) Overview of the TPP-based method for optimizing the nerve cell growth in the NCMC microchannels and its application.

The differentiation process of cells with neurobiological properties generally lasts 7 to 21 d (Fig. 1A). For example, the differentiation cycle of primary hippocampal neurons is 14 to 21 d, and the differentiation cycle of SH-SY5Y cells is 5 to 7 d. One characteristic of cell differentiation is the presence of one or more axons, the length of which extends to at least twice the diameter of the soma [31]. The differential interstitial flow rates responsible for cell positioning and driving cell differentiation at varying speeds are both induced by hydrostatic pressure (Fig. 1B). Furthermore, specific hydrostatic pressure gradients were created at both ends of microchannels with different dimensions to mimic microfluidic effects at various flow rates. Building on this groundwork, on-chip cell culture experiments were conducted. By adding different volumes of culture fluid to the inlet cistern and outlet cistern, a directional fluid force environment from the inlet to the outlet is constructed. By maintaining the pressure difference between the two cisterns in a stable target range, different fluid forces are applied to the axons, prompting the discrete growth of cells along the intended direction.

The cell experiment of NCMC is carried out in 6 steps, as shown in Fig. 1C. Five groups of NCMCs with different dimensions were arranged for cell culture on chip. Poly-L-lysine and collagen were utilized to provide the necessary adhesion conditions for cell growth. After seeding the culture medium and cells, the cells are accurately positioned at the target location because the liquid flow direction points to the outlet. Induction throughout the differentiation process is achieved by applying a greater pressure difference between the outlet and inlet. The fabrication of NCMC provides a platform for differentiated nerve cells to achieve discrete oriented growth of nerve cells. Furthermore, the growth status of fully differentiated nerve cells on NCMC provides negative feedback for the update of NCMC’s structural parameters. Through the closed-loop feedback composed of fabrication of NCMC and on-chip cell growth behavior, NCMC that reaches the target growth state can be accurately constructed. This kind of NCMC can not only be used in the study of axonal injury and regeneration, but also provide a fluid force-driven platform for the study of discrete directional deformation of neurons.

### The NCMC fabrication

**Fig. 2.**
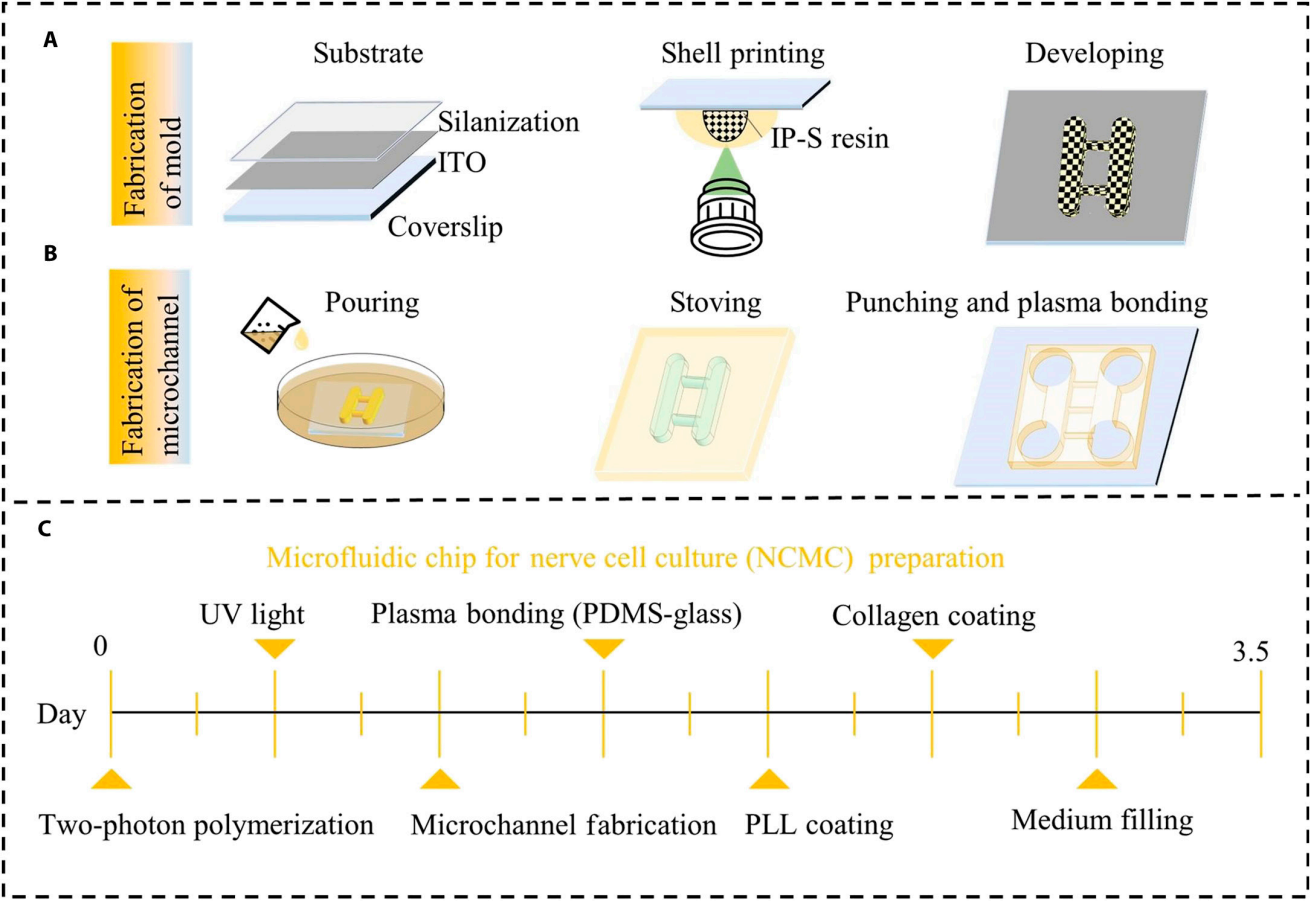
Fabrication processes of the NCMC. (A) Fabrication steps of the NCMC mold based on TPP printing. (B) Fabrication steps of the NCMC based on the NCMC mold. (C) Specific timeline for the NCMC preparation.

**Fig. 3.**
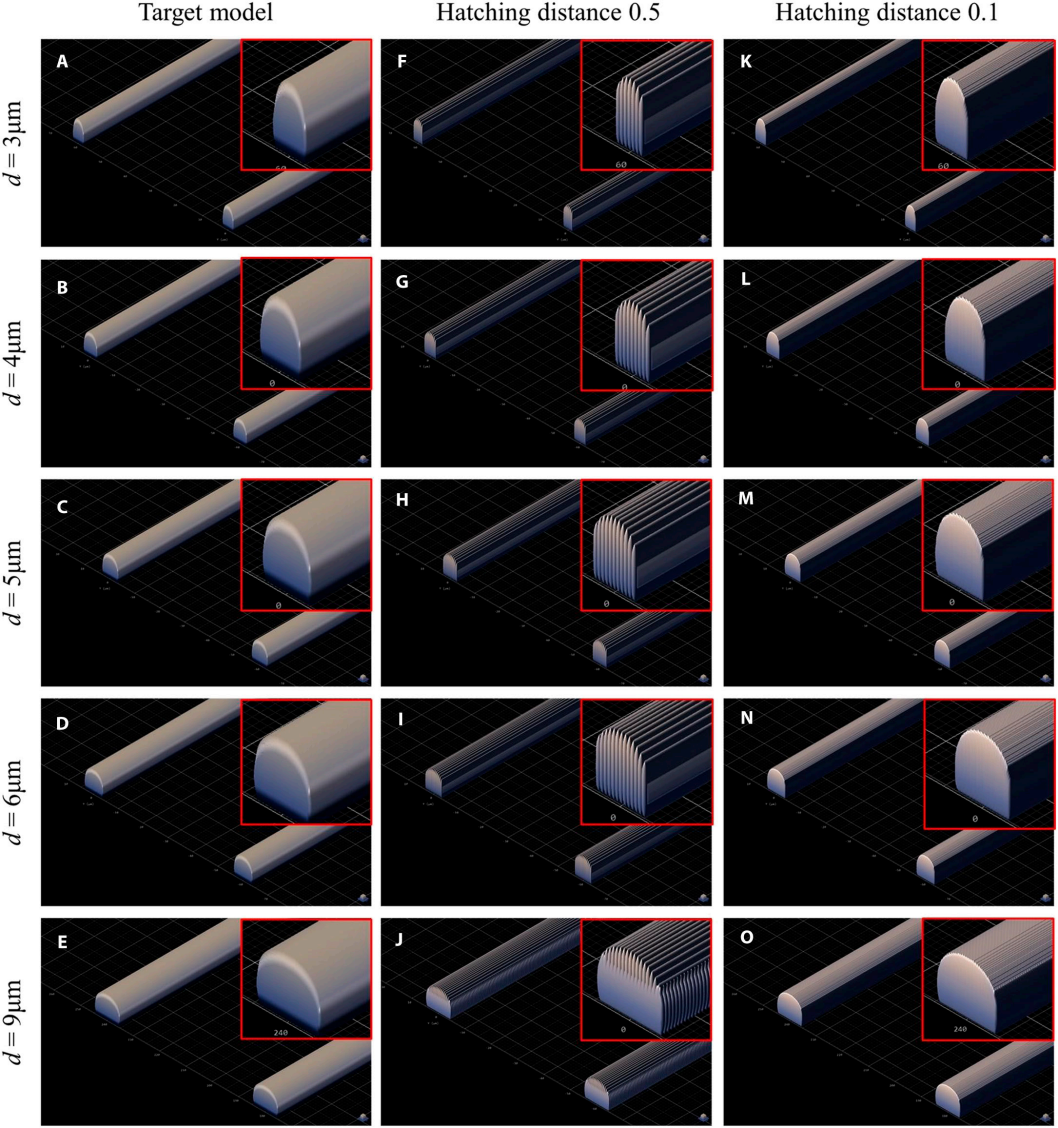
The Describe model based on TPP printing for molding the microfluidic chip with 3D micro-topography (upper right-side view). (A to E) Molds with microchannel width are 3, 4, 5, 6, and 9 respectively. (A) to (E), (F) to (J), and (K) to (O) respectively represent the Target model preview, the model preview with hatching distance (printing resolution) of 0.5, and the model preview with hatching distance of 0.1.

The NCMC consists of a PDMS plate with two open cavities and a 35mm × 35 mm coverslip. The NCMC fabrication is mainly divided into the fabrication of mold and the fabrication of microchannel, as shown in Fig. 2A and B. Prior to all, a 25 mm × 25 mm × 0.7 mm coverslip is used as the printing substrate, and the surface is coated with an ITO layer of 18 ± 6 nm. Before printing, the cover glass was pre-treated with silanization to enhance the adhesion between the photoresist and the substrate and prevent the formed photoresist from falling off together with the PDMS during degumming. The TPP printing and exposure of the mold are completed simultaneously. After developing, the PDMS plate can be obtained by graphic transfer and degumming. PDMS plates and coverslips were plasma treated at an absolute oxygen pressure of 120 Pa (40 ml/min), 40 W, and 30 s (TePla 100 Plasma System, PVA). The PDMS plate was gently pressed to make it fit tightly with the cover glass to form a NCMC with a closed bottom. Figure 2C shows the specific timeline for the NCMC preparation. Differing from the conventional method of making microfluidic chips through 2D photolithography printing, the 3D mold is generated using TPP printing to print IP-S photoresist in 25× ITO shell mode. This technology allows for the rapid and precise manufacturing of micron-level shapes in the Z-direction, offering a high degree of customization. Figure 3 shows the Describe models of different dimensions based on TPP printing for molding the microfluidic chip with 3D micro-topography. It can be observed that when the printing accuracy is accurate to the hatching distance of 0.1, the surface smoothness of the models is basically consistent with the target models.

Taking 2D laser direct writing printing (μPG 101, Heidelberg) and TPP printing (Photonic Professional GT2, Nanoscribe) as representatives, the steps and time used to produce target NCMC by 2D lithography and 3D lithography were compared, as shown in Tables 1 and 2. Compared with 2D laser direct writing printing, TPP printing omits the step of making masks, greatly improving production efficiency and reducing costs. To provide a more visual demonstration of the efficiency of TPP printing, target molds were created using both 2D laser direct writing printing and TPP printing, as shown in Table 3. Within 12 h, the number of target molds printed by TPP is 3 times that of 2D laser direct writing printing. Furthermore, the time required for 2D laser direct writing to produce masks of various dimensions is compared in Table 4. Notably, as the mask pattern becomes larger, the printing time increases significantly. When the resolution of the mask pattern reaches 20, 000 × 20,000 μm, the printing time takes about 2.6 h, resulting in a substantial increase in production time and associated costs.

**Table 1.** Steps for printing microfluidic chips based on 2D lithgraphy (2D laser direct writing) and 3D lithography (2PP)

	Mask substrate preparation	Mask exposure/ printing	Mask development	Chemical corrosion treatment	Alcohol cleaning	Mold substrate surface treatment	Glue coating	Soft bake	Mold exposure/ printing	Post exposure bake	Mold development	Hard bake/ exposure	Graphic transfer	Degumming
2D laser direct writing [45]	✓	✓	✓	✓	✓	✓	✓	✓	✓	✓	✓	✓	✓	✓
2PP [46]	-	-	-	-	-	✓	-	-	✓	-	✓	-	✓	✓

**Table 2.** The time of each step to fabricate microfuidic chips based on 2D laser direct writing and 2PP. (Mold size X/Y/Z: 20,750 μm × 2,100 μm × 100 μm)

	Mask substrate preparation	Mask exposure/ printing	Mask development	Chemical corrosion treatment	Alcohol cleaning	Mold substrate surface treatment	Glue coating	Soft bake	Mold exposure/ printing	Postexposure bake	Mold development	Hard bake/ exposure	Grafic transffer	Degumming
2D laser direct writing	-	2.5 h	<10 min	1 min	<1 min	30 min	10 min	12 min	10 min	10 min	10 min	6 min	1.5 h	2 h
2PP	-	-	-	-	-	-	-	-	1.25 h	-	10 min	6 min	1.5 h	2 h

**Table 3.** Number of target molds (size X/Y/Z: 20,750 μm × 2,100 μm × 100 μm) produced within 12 h based on laser direct writing and 2PP

	Laser direct writing (2D lithography)	2PP (3D lithography)
Number	2	6

**Table 4.** Time required to fabricate masks with different sizes based on 2D laser direct writing

	15μm × 15μm × 100μm	4,075 μm × 3,100 μm × 100 μm	20,750 μm × 21,000 μm × 100μm
Laser direct writing (*μ*PG 101)	10 min 15 s	12 min 37 s	2.6 h

The NCMC adopts the classic “Campenot” design [32], but the internal structure of the microchannel is different. Studies have shown that compared with microchannels with square cross-sections, cells in microchannels with arcuate cross-sections have stronger attachment and can provide more possibilities for changing shape in response to biological stimuli [29]. Therefore, the internal structure of the microchannel adopts an arcuate cross-section design. Referring to the “Anne platform” proposed by Noo et al. [33], the 4 cisterns of NCMC are symmetrically distributed (cylindrical, 6 mm deep, 6-mm bottom diameter). Two cisterns on the same side are connected by the soma chamber or the axon chamber (100 μm deep, 6 mm long, 1.5 mm wide). There are 119 microchannels distributed in parallel between the soma chamber and the axon chamber. Considering the minimum thickness of the soma before and after differentiation, the maximum height of microchannel top surface is designed to be 3 μm. In addition, the microchannel width needs to consider the changes in cell morphology before and after differentiation to avoid soma entering the microchannel and causing blockage of the microchannel or mixing into the axon chamber.

Extracellular matrix proteins such as collagen and fibrin can provide support for cell adhesion, promote cell migration and positioning, and can also affect cell differentiation by providing specific chemical and physical environments. Poly-L-lysine is a synthetic polypeptide widely used in tissue engineering, which can enhance the adhesion between cells and other components in the culture medium or cell culture medium, and promote cell adhesion and growth. The bottom of NCMC was covered with poly-L-lysine at a concentration of 0.01 mg/ml for 2 h, followed by washing it 3 times with deionized water. Then, the bottom surface was treated with a collagen solution at a concentration of 2 μg/cm^2^ and placed in a refrigerator at 4 °C overnight.

To avoid air bubbles in the microchannels, it is necessary to use a pipette to repeatedly extract and inject to force the solution to fill the microchannels before cells seeding [34,35]. The NCMCs filled with culture medium can be stored in the incubator for 2 days before use.

### Cell culture and differentiation

SH-SY5Y cells express multiple nerve cell-specific markers, can differentiate into cells with neuron-like characteristics, and are widely used in neuroscience research, especially in the neuroscience research [30,36,37]. SH-SY5Y cells were cultured in Dulbecco’s modified eagle medium containing 10% fetal bovine serum (FBS, Gibco) and 1% penicillin-streptomycin (P1400, Solarbio). The Petri dishes were kept in the incubator for 48 h with 5% CO_2_ and 37 °C. After removing all the culture medium, cells adhered to the Petri dish bottom were washed with PBS 3 times and detached from the dish using trypsin (Biosharp). Then cells were collected and centrifuged in a test tube, the pelleted cells were suspended in the Dulbecco’s modified Vulture medium containing 3% FBS before fluid induction. Cell suspensions with different densities of 3 × 10^6^ cells/ml and 1.5 × 10^6^ cells/ml were prepared for experiments. Cell differentiation was induced by RA (R2625, Sigma). The concentration of RA in the differentiation medium was 10 μmol/l.

FBS provides essential nutrients, growth factors, and adhesion factors while safeguarding cells against oxidative damage and apoptosis. However, for SH-SY5Y cells, it can suppress cell proliferation and differentiation. Therefore, during the experimental differentiation phase, the proportion of FBS in the culture medium is gradually reduced from 3% to 1% to optimize the cells’ differentiation potential.

Discrete directional growth of axons within NCMC was observed and quantified using an optical microscope (Olympus IX83, Olympus Instrument Inc.). The differentiation process was extended to 21 d, and no further morphological changes were observed.

### Simulation analysis

To analyze the complex variations in the local flow velocity at the entrance region of the microchannel, fluid velocity simulations inside the microchannel were performed using COMSOL software. As the pressure at both ends of each microchannel is entirely the same, only one microchannel is displayed for analysis. The simulation model was designed by SolidWorks and imported into COMSOL. The model was defined on the X–Y plane. The microchannel entrance is directed along the y-axis, directing towards the outlet, in response to the pressure gradient induced by static hydraulic pressure. The liquid chamber was considered inelastic, and the laminar flow was considered incompressible.

### Quantification of the cell viability

The Cell-laden microfluidic chip was cultured at 37 °C and 5% CO_2_. Medium was added every 12 h to maintain the target pressure differential between the inlet and outlet. Live cells and dead cells were stained with fluorescent staining reagents Calcein AM (green fluorescence) and propidium iodide (PI, red fluorescence) stains (Molecular Probes, USA), respectively, and the observation and analysis were completed using ImageJ software within 12 h after the staining was completed.

### Statistical analysis

All values were represented as the mean ± standard deviation (SD). All experiments were performed with *n* = 3 or greater.

## Results

### Microchannel length selection

**Fig. 4.**
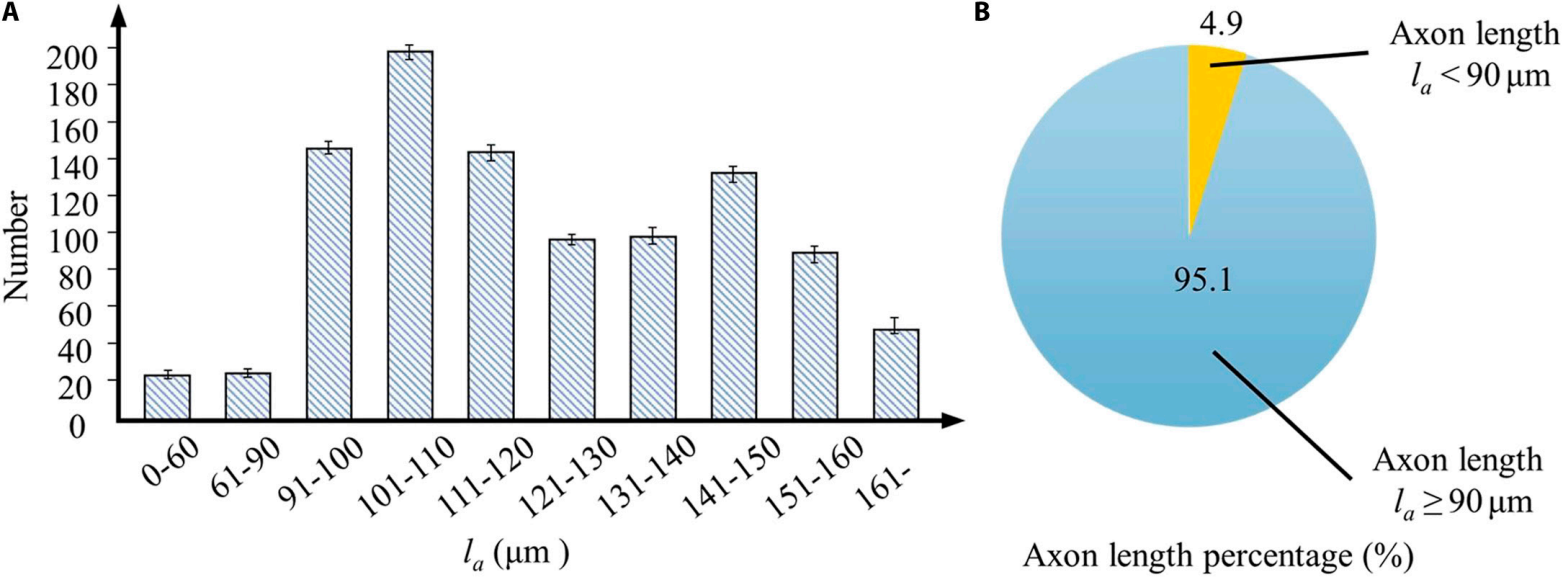
Axon length statistics in Petri dishes with no obstacle. (A) Axon length statistics in different zones in Petri dishes. (B) Percentage of axons with *l_a_* < 90 μm and *l_a_* ≥ 90 μm.

A sufficient number of SH-SY5Y cell samples were cultured in a petri dish to observe and quantify the axon growth of cells. The axon lengths of 1,000 cell samples predominantly fall within the range of 0 to 330 μm, with a concentrated distribution ranging from 60 to 160 μm. These axon lengths can be divided into 10 regions: 8 segments with a 10-μm interval within the concentrated distribution range (60 to 160 μm), as well as two additional regions below 60 μm and above 160 μm, as shown in Table 5. The statistical analysis of cell counts within each length region is illustrated in Fig. 4A. The proportion of cells with axon lengths greater than or equal to 90 μm is approximately 95.1%, while the proportion of cells with lengths below 90 μm is approximately 4.9% (Fig. 4B). To ensure an adequate number of axons in the axon chamber for subsequent functional experiments, the length of the microfluidic channel should not exceed 90 μm. Simultaneously, to ensure complete cell differentiation, the relative distance between the soma chamber and the axon chamber should be as far apart as possible. Therefore, the microchannel length is set to 90 μm.

**Table 5.** Axon length (*l_a_*) of SH-SY5Y cells samples (*n* = 1,000) in Petri dishes

Zone (μm)	0–60	61–90	91–100	101–110	111–120	121–130	131–140	141–150	151–160	161–
Number	24	25	144	198	143	99	101	133	88	45

### Discrete and directional growth of axons

**Fig. 5.**
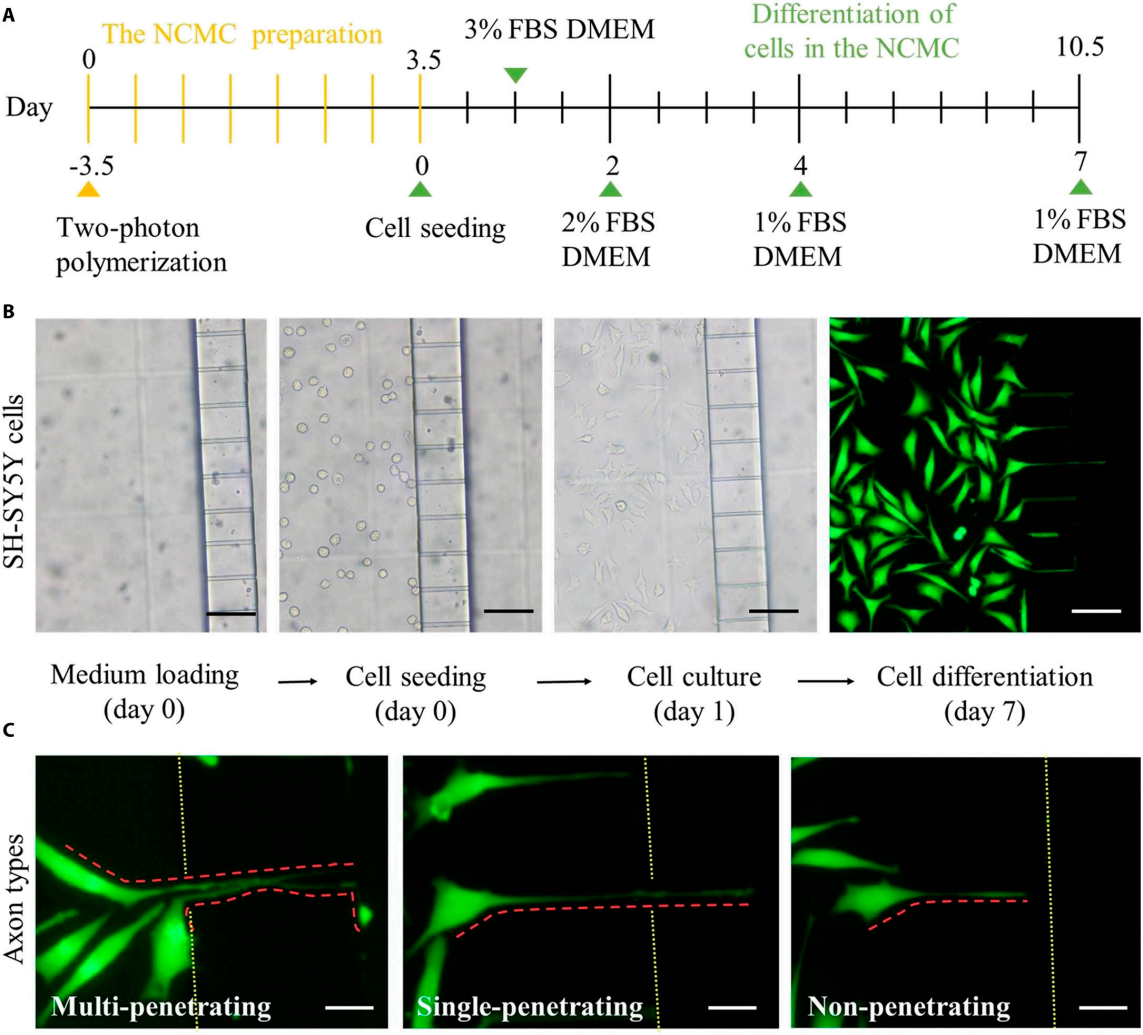
Cell culture and differentiation experiments in the NCMC. (A) Specific time schedule for cell differentiation experiment in the NCMC. (B) The optical microscopy images of medium loading, SH-SY5Y cell seeding, cell culture, and cell differentiation in the NCMC based on the optical microscope. Scale bars: 90 μm. (C) Fluorescent images of three axon types through microchannels of the NCMC after Calcein AM and PI treatment. The red dotted line marks the shape outline of the axon. Scale bars: 25 μm.

In neurobiological research, the discrete and directional growth of nerve cells is widely used in nerve injury and regeneration experiments [38]. In addition to constructing functional artificial biomicromodules to simulate neural tissue in vivo, the microfluidic chip for directional growth of neural cells is also an experimental platform for drug testing, cell culture, and cell behavior research. SH-SY5Y cells achieve discrete and directional growth throughout the microchannels in NCMCs, as shown in Fig. 5. In Fig. 5A, the time consumption of the key experimental steps is represented by segments of varying lengths. The yellow and green segments correspond to NCMC preparation and on-chip cell differentiation experiments, respectively. As depicted in Fig. 5A, it is evident that chip preparation takes approximately one-third of the total experiment duration. The reduction in chip preparation time significantly enhances the overall efficiency and repeatability of the experiment.

During the entire experimental process, the optical microscopy images of medium loading, cell seeding, and cell culture in the NCMC under the bright field were recorded, as shown in Fig. 5B. Cells began to adhere to the wall 0.5 h after seeding and completely adhered to the soma chamber bottom 4 h after seeding. Twenty-four hours after implantation, the cells showed polarity changes, the soma became smaller, and multiple short neurites extended out, showing significant characteristics in the early stage of differentiation. At the end of differentiation, the soma narrows and the axon width is only 1 μm, making it difficult to observe under bright field conditions. Therefore, cells were treated with Calcein AM and PI, and differentiation images of the cells at 10× were recorded by fluorescence microscopy. At d7, multiple axons extended into the microchannels. Somas are firmly positioned on the side of the soma chamber, and only axons pass through the microchannel and extend into the axon chamber. On this basis, axons in microchannels can be divided into three types according to their growth characteristics: nonpenetrating, single-penetrating, and multiple-penetrating, which respectively represent axons not penetrating the microchannel, single penetrating microchannel, and multiple penetrating microchannel, as shown in Fig. 5C.

### Optimization of flow velocity

**Fig. 6.**
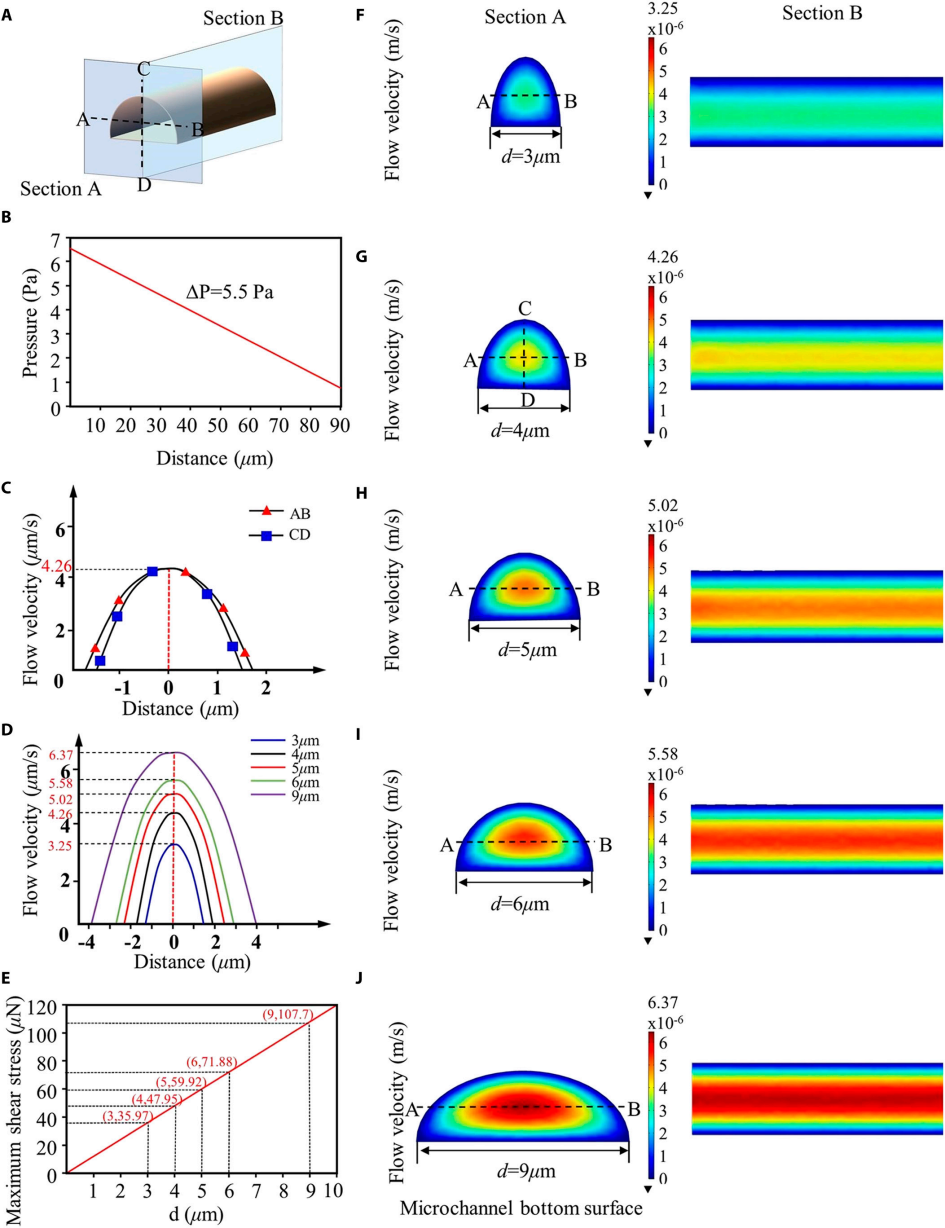
Simulation result by COMSOL Multiphysics. (A) Sections A and B are selected on the microfluidic channels. AB and CD are the horizontal and vertical lines passing through the center point on section A. (B) Variation of pressure in the microchannel with distance from the inlet. (C) Variation of flow velocity on AB and CD in the microchannel with d = 4 μm. (D) Flow velocity on AB in the NCMCs with different widths. (E) Maximum shear stress in the microchannels with different widths. (F to J) Simulation of the flow in the microchannels of NCMCs with different widths.

The fluid around the cells is in direct contact with the soma and axons, directly affecting the differentiation behavior of the cells. Complex changes in local flow velocity in the microchannel entrance area may cause abnormal axonal differentiation. To reduce damage to axons caused by velocity changes within the extreme distance, attempts were made to explore fluid velocity and shear force changes in microchannels of different widths. Based on the model in Simulation analysis, COMSOL software was used to analyze the fluid velocity distribution and dimensions within microchannels of different widths, as shown in Fig. 5. Since the instantaneous change of flow velocity affects axon growth mainly in the inlet area of the microchannel, the entrance section (Section A) and the longitudinal section (Section B) are selected to express the local flow velocity dimensions and distribution in the microchannel, as shown in Fig. 6A. Based on the research of Eide et al. [39], the average pressure in the brain environment of patients with general CNS diseases is about 5.5 pa, so 5.5 pa is selected as the unified initial pressure difference at both ends of the five groups of microchannels with different widths (Fig. 6B). The fluid flows from the inlet to the outlet and the pressure decreases, driving the axon to extend and grow in the direction of the outlet until it penetrates the microchannel. Section A and Section B are semielliptical and rectangular respectively, as shown in Fig. 6F to J. It can be seen from the simulation results in Fig. 6C and D that the maximum velocity values in the 5 groups of microchannels are all located at the center point of Section A. Taking this center point as the starting point, selecting AB and CD to analyze the changes in fluid velocity, it was found that the velocity decreased symmetrically toward the microchannel wall in a parabolic shape. However, there are obvious differences in the range of velocity changes in the 5 groups of microchannels. As the width of the microchannel *d* increases, the maximum velocity in the microchannel gradually increases. When *d* = 9μm, the maximum velocity in the microchannel is the largest, reaching 6.37 μm/s. In contrast, when *d* = 3 μm, the maximum velocity in the microchannel is the smallest, 3.25 μm/s, which is about half of the maximum velocity in the microchannel with *d* = 9 μm. Combining the research of Wang and Begehr, the (maximum) shear force relationship is calculated [40,41]:

$$\tau =-\frac{\Delta P}{2l}R=-\frac{3\pi d\Delta P}{2l\left(2\pi d+24\right)}$$ (1)

when *l* is the tube length. *R*is the hydraulic radius of tube. Therefore, the fluid shear stress should increase linearly with the microchannel width, as shown in Fig. 6E. When *d* = 3 μm, the maximum shear stress in the microchannel is the smallest, about 35.97 μN.

The simulation results of Fig. 6 show that under the uniform brain environment pressure common in patients with CNS diseases, microchannels with smaller width can effectively slow down the local flow velocity and reduce the variation range of flow velocity. Therefore, microchannels with smaller width are more conducive to reducing damage to axons within the extreme distance and effectively protecting the differentiation process of axons.

### Chip dimensions match cell growth properties

**Fig. 7.**
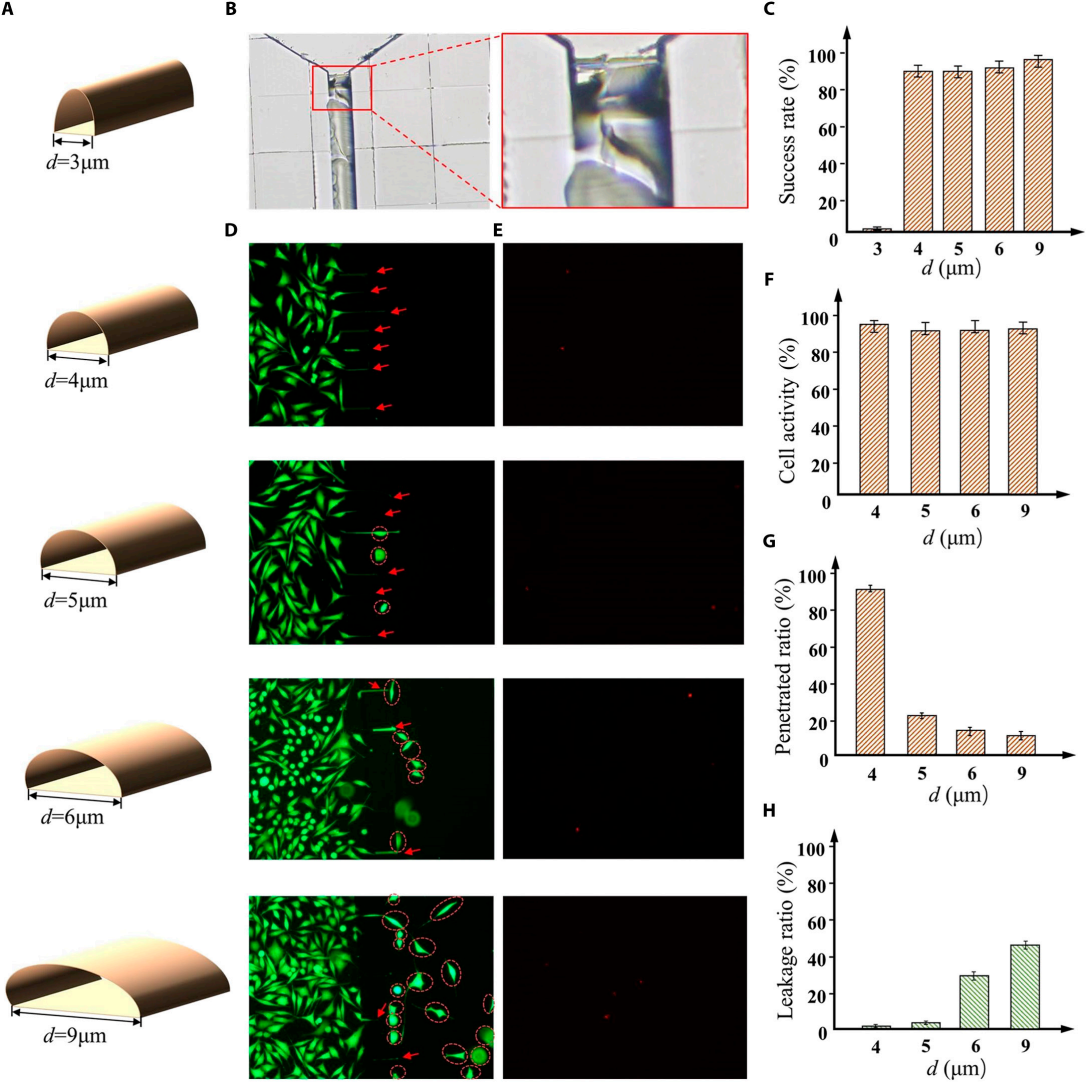
SH-SY5Y cell culture experiments in the microchannels of NCMCs with different widths. (A) The NCMCs with microchannel width are 3, 4, 5, 6, and 9 respectively. (B) The PDMS block broke into the gap between the soma chamber and the axon chamber. (C) The success rate of NCMC fabrication with different microchannel widths. (D) Fluorescence images of cells in the NCMC with different microchannel widths. Red dotted circles encircle leaking cells. Red scissors indicate axons passing through the microfluidic channel. (E) Images of dead cells in the NCMC with different microchannel widths. (F) Cell activity in the NCMCs with different widths. (G) Penetrated ratio: the proportion of microchannels penetrated by axons relative to the total number of microchannels. (H) Leakage ratio: the proportion of cells entering the axon chamber through microchannels relative to the total cell count.

As described in Experimental design, we performed preliminary experiments applying the TPP-based microfluidic chip design and fabrication method to the directional growth of SH-SY5Y cells. Cells were cultured and differentiated in NCMCs with different microchannel widths, as shown in Fig. 7. SH-SY5Y cells are about 10 to 25 μm wide before differentiation, and the soma width is about 9 to 15 μm after differentiation. Therefore, to minimize cell leakage into the axon chamber, NCMCs with 5 different widths (*d* = 3, 4, 5, 6, and 9 μm) were selected and used for cell discrete directional growth experiments (Fig. 7A). The microchannel height is designed to be 3um. The soma chamber was implanted with a sufficient number of cells to ensure that the axon penetration ratio was not affected by insufficient cell numbers. By adding 65 μl and 10 μl culture medium to the inlet and outlet cisterns respectively, a directional fluid force environment from inlet to outlet was constructed. Figure 7B shows the failed fabrication of NCMC with *d* = 3 μm. Because the microchannel width is too small, the cured PDMS is difficult to demold. Therefore, the PDMS plate is easily broken in the gap between the soma chamber and the axon chamber during degumming, which increases the complexity of NCMC fabrication. When *d* = 3 μm, the success rate of NCMC fabrication is approximately 2%. Therefore, cell experiments were only performed on the other four groups of NCMCs. Compared to NCMCs with *d* = 3 μm, the success rates of NCMCs with *d* = 4, 5, and 6 μm all remained at around 90% (Fig. 7C), and NCMC with *d* = 9 μm had a fabrication success rate of approximately 95%, demonstrating high repeatability. This is because, when *d* = 9 μm, the wider microchannel allows for easier detachment of the cured PDMS from the mold without residue.

The fluorescent images of live cells in NCMCs with different microchannel widths are shown in Fig. 7D. Additionally, fluorescent images of dead cells were also detected (Fig. 7E). Results from the live-dead cell staining experiment indicate that cell viability within the 4 NCMC groups is relatively consistent and remains stable at approximately 98% (Fig. 7F). It was observed that with an increase of microchannel width, the number of cells leaking into the axon chamber gradually increased, while the number of axons passing through and remaining in the microchannel decreased progressively (Fig. 7G and H). This phenomenon can be attributed to the robust migratory capability of the cells, enabling them to deform and traverse through channels slightly narrower than their own width. Therefore, when the microchannel width is 5 to 9 μm, part of the SH-SY5Y soma can pass through the narrow microchannel and enter the axon chamber. It is worth noting that when *d* = 4 μm, the microchannel width is too narrow for the soma, so the cells can achieve 0 leakage into the axon chamber, while the axon still maintains the relatively highest penetration ratio.

The results indicate that the TPP-based microfluidic chip design and fabrication method can explore the most suitable geometric parameters for the microfluidic chip structure corresponding to SH-SY5Y cells. These structural geometric parameters of the microfluidic chip can effectively match the growth characteristics of SH-SY5Y cells, achieving a high proportion of axon penetration on the chip, while ensuring high cell activity and 0 leakage of cells into the axon chamber.

## Discussion

The efficient and customizable method of microfluidic chip design and manufacturing holds marked application value for the development of functional neural microfluidic chips and their associated biomedical research. Various additive manufacturing technologies have been used to construct functional neural microfluidic chips in vitro [42–44]. As one of the most important additive manufacturing technologies, TPP technology can achieve efficient and high-precision micron-level printing based on biocompatible materials, becoming a powerful tool in the fields of neurobiology, medicine, and bioengineering. In this paper, we propose a TPP-based microfluidic chip design and fabrication method to optimize the discrete and directional growth of neural cells. To minimize the damage to axons and effectively protect the differentiation process of axons, the internal structure of microchannel with 3D micro-topography was selected based on the “Campenot" structure and "Anne platform" by considering the adhesion properties of cells. In terms of the fabrication of 3D micro-topography, a mold fabrication method based on TPP printing is proposed to achieve rapid printing and simultaneous exposure of molds of different dimensions with micron-level precision. The morphological changes during the differentiation process of cells with neural differentiation properties and their differentiation cycles were compared and analyzed to select the appropriate cell type for verifying the optimality of the manufactured NCMC. The dimensions of microchannels within the NCMC were then tailored to align with the biological characteristics of the chosen cell type. The fabrication success rate of NCMC with various microchannel dimensions was examined to identify effective and feasible microchannel dimensions. On this basis, the comparative experiments of directional axon growth were conducted within NCMCs of different microchannel dimensions using the selected cell type. These experiments yielded the optimal discrete and directional growth results for the chosen cell type, along with their corresponding microchannel geometric parameters. This cellular experiment, as an initial endeavor, serves as evidence of the effectiveness and reliability of the proposed TPP-based design and fabrication method. Experimental results show that this method can fully match the growth characteristics of cells, construct neural microfluidic chips with high efficiency and high precision, and provide a new solution for precise regulation of biological growth at the microscale.

In the future, this method is expected to be used in the development of in vitro functional neural microfluidic chips with more complex structures, such as functional verification of nerves within multilayer, nested or composite structures, quantitative analysis of axonal growth and cell-cell interaction studies. The fluidic field within microfluidic chips can drive changes in cellular behavior, influencing the process of constructing biomimetic tissues in vitro. This holds important importance for the development of more complex in vitro functional neural microfluidic chips. By investigating the relationship and mechanisms between fluidic forces and cell growth parameters in microfluidic chips, we can precisely regulate cell growth and construct target biomimetic tissues with greater accuracy. However, there are still some technical challenges that require more attention. With the increasing functional requirements of neural microfluidic chips, the limited availability of materials for TPP printing technology is a concern. Additive manufacturing technologies with an expanded material palette may offer a potential solution to this issue. To enhance the effective modulation of the functional properties of neural microfluidic chips after in vitro cultivation, further quantifying the growth mechanisms of cells in fluid-driven environments becomes a potential solution. Therefore, we will further explore and analyze the dynamics and kinematics of fluid-driven cell growth, as well as the action mechanism of mechanical stimulation on cells.

## Data Availability

The data are freely available upon request.
